# Triggered Release from Thermoresponsive Polymersomes with Superparamagnetic Membranes

**DOI:** 10.3390/ma9010029

**Published:** 2016-01-06

**Authors:** Oliver Bixner, Steffen Kurzhals, Mudassar Virk, Erik Reimhult

**Affiliations:** 1 Institute for Biologically Inspired Materials, Department of Nanobiotechnology, University of Natural Resources and Life Sciences, Vienna, Muthgasse 11, Vienna 1190, Austria; oliver.bixner@boku.ac.at (O.B.); steffen.kurzhals@boku.ac.at (S.K.); mudassar.virk@boku.ac.at (M.V.); 2 School of Materials Science and Engineering, Centre for Biomimetic Sensor Science, Nanyang Technological University, 50 Nanyang Drive, Singapore 637553

**Keywords:** superparamagnetic iron oxide nanoparticle, thermoresponsive polymer, magneto-polymersome, magneto-thermal release, calcein assay

## Abstract

Magnetic polymersomes were prepared by self-assembly of the amphiphilic block copolymer poly(isoprene-*b*-*N*-isopropylacrylamide) with monodisperse hydrophobic superparamagnetic iron oxide nanoparticles (SPION). The specifically designed thermoresponsive block copolymer allowed for efficient incorporation of the hydrophobic nanoparticles in the membrane core and encapsulation of the water soluble dye calcein in the lumen of the vesicles. Magnetic heating of the embedded SPIONs led to increased bilayer permeability through dehydration of the thermoresponsive PNIPAM block. The entrapped calcein could therefore be released in controlled doses solely through exposure to pulses of an alternating magnetic field. This hybrid SPION-polymersome system demonstrates a possible direction for release applications that merges rational polymersome design with addressed external magnetic field-triggered release through embedded nanomaterials.

## 1. Introduction

Amphiphilic diblock copolymers with hydrophilic volume fractions around 30%–50% v/v spontaneously self-assemble into vesicular spherical structures with bilayer membranes [1,2,3,4]. Such polymersomes have received rapidly increased attention since they offer an improved and designable alternative to liposomes for encapsulation and release applications such as delivery of bioactive compounds *in vivo* and *in vitro* [5,6,7,8,9]. A large aqueous lumen can efficiently transport hydrophilic substances; the tailorable amphiphilic membrane can host hydrophobic compounds in the membrane interior of higher molecular weight and more complex composition than lipid membranes can [10]. Improved mechanical stability is readily achieved by amplifying the respective block weights at constant volume ratios without compromising assembly behavior [11]. The high robustness correlates with a drastically lowered permeability and thereby lowered passive leakage of transported cargo. However, high stability and low permeability pose a challenge for release applications that require rapid and efficient release at the target site. Considerable effort has been devoted to the implementation of stimuli responsive vesicles that burst or decompose upon exposure to environmental changes such as temperature, pH or subcellular localization [7,8,9,12,13]. This is achieved by including stimuli-responsive units into the vesicle membrane that break down the membrane integrity upon the stimulus.

A particularly appealing approach is to include membrane components that respond to external field triggers and therefore can cause release without relying on uncontrolled changes in the surrounding environment. The external field trigger is typically light or magnetic fields, with which polymers have weak interactions but for which inorganic nanomaterials can serve as strong enhancers [9,14,15,16,17,18,19,20,21,22,23,24,25,26]. Field-triggered release offers decisive advantages such as control over the release profile by switching on and off the stimulus and release in locations where direct changes to the environment through e.g. change in pH or heating are not possible due to lack of direct access.

Superparamagnetic iron oxide nanoparticles (SPIONs) actuated by alternating magnetic fields are highly suitable enhancers for biomedical applications due to the low susceptibility of tissue to magnetic fields; they offer the additional advantage of being biocompatible through hydrolytic degradation into constituent ions that can be recycled by the body [27]. Release from thermally responsive PEG-liposomes using embedded SPIONs for magnetic heating has been shown; it was furthermore demonstrated that embedding of the nanoparticles in the membrane interior to directly affect the permeability is greatly advantageous [28,29]. SPIONs have also been incorporated into various polymeric assemblies [15]. Recent studies on the co-self-assembly of high molecular weight (10–22 kDa), nonresponsive PAA-*b*-PS block copolymers with SPIONs demonstrated that nanoparticle distribution within the resulting assemblies can be controlled by solvent selection; diverse structures such as micelles, core-shell structures and polymersomes incorporating dense and diffuse layers of SPIONs could be assembled [16].

**Scheme 1:**
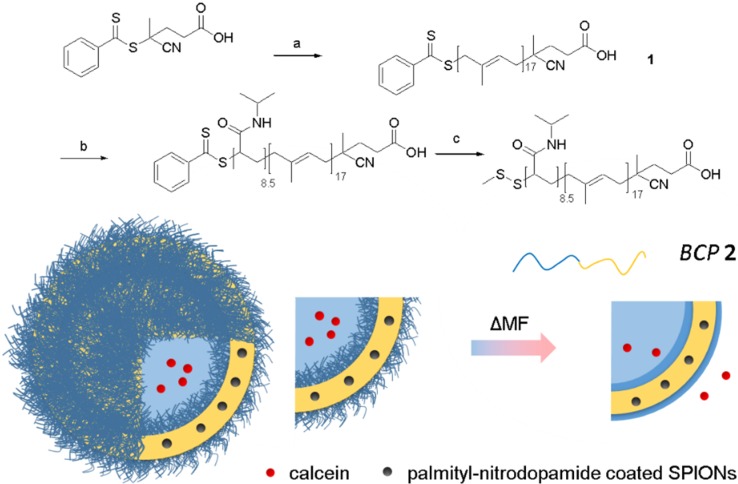
Preparation of thermoresponsive block copolymer by sequential RAFT polymerization, **a** isoprene, AIBN, THF, **b** *N*-isopropylacrylamide, AIBN, dioxane, **c** *S*-methyl methanethiosulfonate, (2-dimethylamino) ethylamine. The schematics show polymersome structure and triggered release. Orange line: hydrophobic block (polyisoprene); blue line: hydrophilic block (poly(*N*-isopropyl acrylamide); black dots: palmityl-nitrodopamide coated iron oxide nanoparticles; red dots: calcein. The triggered release of the calcein is accomplished by an alternating magnetic field that heats the embedded nanoparticles. Increased temperature dehydrates the PNIPAM block and increases polymersome membrane permeability.

The most commonly employed diblock copolymers used for the preparation of thermoresponsive vesicles are poly(*N*-isopropylacrylamide) (PNIPAM) based [30]. Applications that make use of PNIPAM as the hydrophobic block are manifold [8], but they display a release of encapsulated compounds below the lower critical solution temperature (LCST), *i.e.*, the vesicles disassemble upon cooling in hypothermia applications [19,20,21,22,23]. It is, however, desirable to develop a design that achieves release of cargo upon heating, since an increase in temperature can be achieved through actuation of nanoparticles by an alternating magnetic field. PNIPAM partly loses its solubility in water upon heating [31]. PNIPAM is therefore suited to demonstrate release at temperatures above the LCST of the hydrophilic block. Partial dehydration of a hydrophilic PNIPAM block should change the block copolymer packing parameter that determines the integrity and thereby permeability of the polymersome membrane (Scheme 1).

Magnetically actuated release of cargo from SPION-loaded block copolymer vesicles has so far only been demonstrated for poly(trimethylene carbonate-*b*-glutamic acid) [24,25] and poly(acrylic acid-*co*-distearin acrylate) [32]. Hydrophobic doxorubicin associated with the membrane of the polymersomes was released by nanoparticle-mediated hyperthermia in these cases [24,25,26,32]. The incorporation of SPIONs in these examples seems to have led to rather undefined structures with aggregated particle clusters in the membrane, which could explain high passive leakage of the membrane-associated drug [24,25]. In addition, although local hyperthermia increased the release rate in agreement with predictions, the polymersome membrane did not undergo a structural phase transition similar to what was demonstrated for the more efficient triggered release from liposomes in response to local hyperthermia [24,25,29]. The slow rate of doxorubicin released from the membrane was therefore only marginally faster than the passive leakage without application of a magnetic field.

Our goal with this work is to demonstrate SPION-loaded polymersomes with low membrane permeability that are able to encapsulate hydrophilic compounds in the lumen and release them by magneto-thermal actuation. Our focus here, compared to previous work, is on using the lumen of the vesicles to encapsulate a high concentration of hydrophilic molecules by a magnetic membrane that shows low passive leakage. Importantly, we also strive to improve the release rate and profile over what has been demonstrated for just hyperthermia affecting the hydration and packing of chains in the membrane core; this can be achieved by achieving a larger structural change in the membrane through dehydration of the hydrophilic block. We therefore report on the synthesis and assembly of the vesicle-forming, thermoresponsive di-block-copolymer polyisoprene-block-poly(*N*-isopropylacrylamide) (PI-*b*-PNIPAM) with controlled embedding of SPIONs stabilized with densely and strongly anchored hydrophobic ligands within the polymersome membrane. An important improvement is the use of nanoparticles with well-defined size, shape and dense hydrophobic ligand shell that is irreversibly grafted; the importance of controlling nanoparticle properties to avoid aggregated nanoparticle-membrane structures and to suppress passive leakage from vesicles has previously been demonstrated for liposomes [29]. In these vesicles, the thermoresponsive polymer PNIPAM serves as the hydrophilic block. Finally, we demonstrate triggered release of calcein encapsulated in the lumen of the polymersomes by magneto-thermal actuation.

## 2. Results

A PI-*b*-PNIPAM block copolymer (BCP **2**) with thermoresponsive volume fractions of 40% v/v was prepared by sequential RAFT polymerization (Scheme 1; for detailed description see Materials and Methods Section and Figures S1–S3). The first step involved polymerization of isoprene at 125 °C to prepare macro RAFT agent **1** with a molecular weight of approximately 1.3 kDa (>90% 1,4-addition). The presence of reactive dithiobenzoate end groups in **1**, which is crucial for the polymerization of the second block, was confirmed by ^1^H-NMR resonances at 7.98, 7.51, 7.37 ppm and characteristic UV/VIS absorptions at 295 nm and 500 nm. Macro RAFT agent **1** was subsequently used for the polymerization of *N*-isopropylacrylamide in dioxane where different PNIPAM block sizes could be obtained by adjusting the monomer-to-macro RAFT ratio. The remaining head group was conveniently cleaved by aminolysis and trapped as disulfide to give the final α,ω-heterobifunctional block copolymer **2** after column chromatography. The synthesis of *N*-palmityl-6-nitrodopamide coated superparamagnetic iron oxide nanoparticles (P-NDA-SPION) with a core diameter of 3.5 ± 0.4 nm was described previously [33].

Vesicle formation of SPION-loaded BCP **2** depended on experimental conditions such as preparation method, temperature, aqueous phase composition and additional energy input (e.g., sonication). Initial attempts to produce loaded vesicles via standard rehydration in Milli-Q/calcein (5 mg/mL; 0.2 µm filtered) or phosphate buffered saline (PBS; 10 mM NaHPO_4_/150 mM NaCl)/calcein solution required improvement because of minimal dispersion of the nanoparticle/BCP **2** film into those phases at ambient conditions. Neither gentle temperature variations nor sonication improved on vesicle formation.

Vesicles of BCP **2** (M_n_ ~ 2300 g/mol, Ð = 1.14, φ (PNIPAM) = 40% v/v) were instead prepared at 1 mg/mL by solvent inversion into ultrapure water and calcein similar to Meier *et al.* for pure polymersomes [34] and Bixner *et al.* for magnetic liposomes [35]. A detailed protocol can be found in the Materials and Methods Section. Typically, BCP **2** (1 mg) and the desired SPION weight fraction (20% w/w) were dissolved in THF (200 µL) and subsequently added dropwise at room temperature into ultrapure water (2 mL) or calcein solution (5 mg/mL; 0.2 µm filtered) under magnetic stirring. THF was evaporated at room-temperature under a constant N_2_ stream for 3 h and the sample was refilled with Milli-Q to the original concentration. The remaining THF concentration in the sample is after 3 h negligible and far below toxic levels (see Section S3; Figure S4).

**Figure 1:**
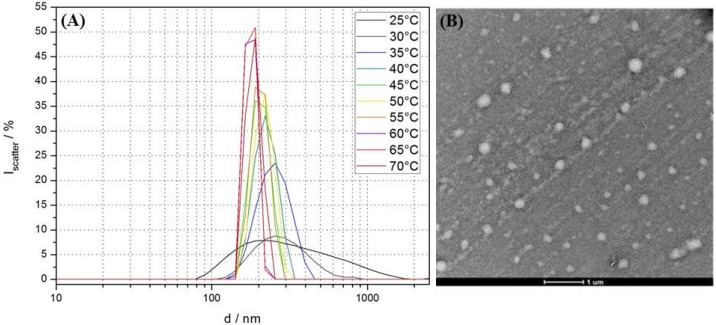
(**A**) Temperature-dependent DLS size distributions at 25–70 °C in 5 °C steps of the crude PI-*b*-PNIPAM assemblies at 1 mg/mL prepared by THF solvent inversion into Milli-Q water; (**B**) TEM after trehalose fixation of the sample shows spherical objects with a similar size distribution as obtained from room-temperature DLS. The lower contrast of the vesicular structures is because water in the lumen of the vesicles is not replaced by trehalose.

Dynamic light scattering (DLS) showed structures with a broad distribution of hydrodynamic sizes of 0.1–1 µm for the turbid as-prepared suspension (Figure 1A). TEM of the same sample showed spherical hollow structures with a size distribution similar to the one obtained by DLS, further supporting successful formation of polydisperse block copolymer vesicles (Figure 1B). Figure 1A also shows the results of temperature-dependent DLS in the range of 25–75 °C in 5 °C steps. During temperature cycling, the initial broad distribution sharpened at 30 °C to a maximum centered at 250 nm. In the range from 35 to 70 °C the hydrodynamic diameters only shifted slightly to approximately 200 nm but steadily increased in intensity to ultimately settle at seven-fold of the initial value at 50 °C. No further change in size distribution up to 70 °C was observed. This result demonstrates the thermoresponsiveness of BCP **2** vesicles with a transition temperature range of 35–50 °C; this is higher than the typical literature value of 32 °C, but an increased LCST and even suppression of the collapse of the coil is expected for low molecular weight PNIPAM in an amphiphilic environment [36].

**Figure 2:**
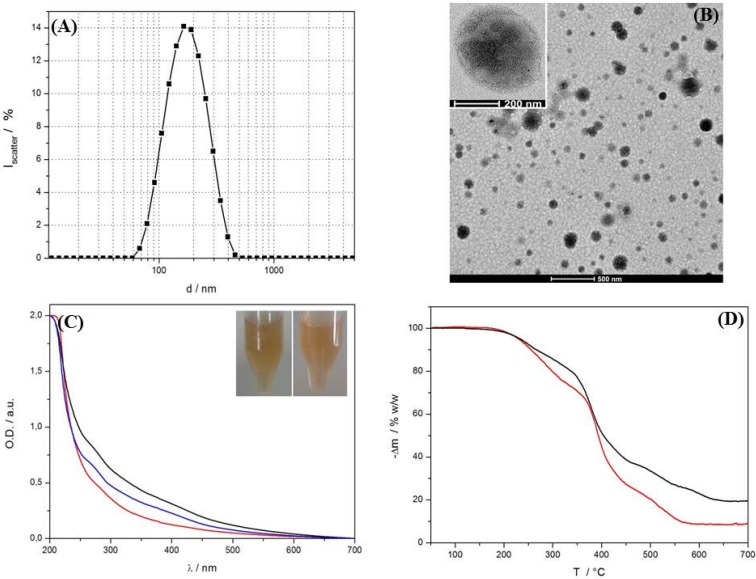
(**A**) DLS size distribution and (**B**) TEM micrograph of calcein-loaded, extruded PI-*b*-PNIPAM polymersomes at 1 mg/mL with 20% w/w 3.5 nm hydrophobic SPION input. Samples were prepared by THF solvent inversion into 5 mg/mL calcein solution to form polydisperse, large polymersomes and subsequent extrusion through 100 nm track-etched polycarbonate membranes after evaporation of the organic solvent. A high SPION content is seen from the high contrast of most vesicles and the cores are directly visualized in the inset (see also Figure S5); (**C**) Optical density curves of extruded PI-*b*-PNIPAM vesicles without nanoparticles (red), SPION loaded polymersomes before (black) and after (blue) homogenization by 10 passes through 100 nm track-etched polycarbonate membranes. The inset shows a digital image of the preparations before and after extrusion; (**D**) TGA curves (20–650 °C) of BCP **2** and BCP **2** extruded with 20% w/w P-NDA-coated iron oxide nanoparticles. Iron oxide content (taking inorganic residue of BCP into account) is estimated to be ~9% w/w, which is significantly higher than described for liposome [28].

Multilamellar large vesicles are of limited use for release applications. Standard methods to enforce unilamellarity and decrease vesicle size are sonication and extrusion, which have been successfully applied to polymersomes [36]. Sonication at constant *T* = 20 °C led to polymer and nanoparticle precipitation. Extrusion through track-etched polycarbonate membranes caused loss of hydrophobic SPIONs and some polymer but did not lead to precipitation. The measured DLS curves and OD^350^ values (Figure 2) of the extruded preparations matched the expected changes based on similar preparations of liposomes, for which the lamellarity is known to be reduced. The solution became clearer, which indicates a reduction in size but primarily a lower fraction of multilamellar vesicles. Multilamellarity is well-known to more strongly increase scattering in the optical range than size. The similar hydrodynamic sizes measured in DLS before and after extrusion also strongly indicate that the major change in polymersome morphology is a reduction in lamellarity. We therefore used extrusion (10×, 100 nm polycarbonate membranes) to create monodisperse unilamellar thermoresponsive polymersomes encapsulating calcein in the lumen, while retaining a high SPION content.

DLS size distributions (159 ± 66 nm) and TEM of extruded SPION-loaded vesicles with encapsulated calcein are shown in Figure 2A,B. The orange-brown suspensions after extrusion were clear as expected for predominantly unilamellar vesicles. More SPION than polymer are lost in the extrusion and for an initial input of 20% w/w 3.5 nm SPION we determined an incorporated weight fraction of around 10% w/w by TGA (rest mass after thermal decomposition relative to total organic content) and UV/VIS spectroscopy (characteristic wavelength at 350 nm) (see Figure 2C,D). A detailed description of the determination of the nanoparticle loading content is provided in the supporting information (Sections S4.2 and S4.3).

The fluorescent dye calcein was encapsulated at self-quenching concentrations. Calcein is water-soluble and serves as an easily quantifiable model compound to investigate passive and magneto-thermally triggered release through the polymersome membrane. Samples were purified from excess dye by size exclusion chromatography over a Superdex 75 FPLC column (GE Life Sciences, Austria) in ultrapure water and fractionated according to UV absorption and refractive index. The purification reduced the sample concentration from the original 2 mg/mL to 0.5 mg/mL. The encapsulated calcein thereby remains at self-quenching concentration while the external calcein is removed.

**Figure 3:**
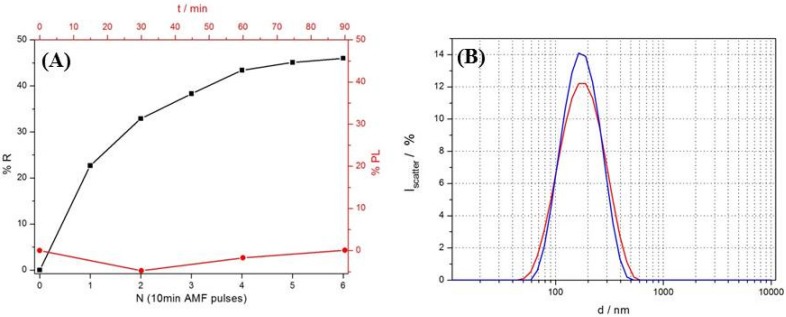
(**A**) Release kinetics of calcein encapsulated in 3.5 nm hydrophobic SPION-loaded PI-*b*-PNIPAM polymersomes. The samples were actuated with 10 min AMF pulses followed by a 5 min cool-down period. 50% release was achieved in less than 1.5 h (black line—squares, left axis). The passive leakage measured at 24 °C during the same period was negligible (red line—circles, right axis); (**B**) The hydrodynamic size distribution of the polymersomes measured before (blue) and after (red) actuation by AMF is almost unchanged, indicating increased permeability without destruction of the vesicles.

Release of encapsulated calcein to the bulk phase was quantified by recording the increase in fluorescence intensity as function of time and membrane actuation on the bulk sample stored in a cuvette. The change in fluorescence intensity was obtained after subtraction of background fluorescence and normalizing to the total fluorescence after disruption of the vesicles by Triton X-100 (Sigma Aldrich, Austria). Magneto-thermal release was triggered by applying an alternating magnetic field (AMF) of variable duration and intensity. The resulting relative increase in fluorescence was compared to the passive release in absence of an alternating magnetic field. The fluorescence resulting from triggered release of calcein from PI-*b*-PNIPAM polymersomes with 3.5 nm SPIONs incorporated in the membrane is shown in Figure 3. The AMF causes heat to dissipate locally from the magnetic cores due to Néel relaxation [27,28]. It was found that only a long pulse duration of 10 min led to significant release. Application of one pulse of 8 min duration triggered only 3% release of entrapped calcein whereas application of one 10 min pulse triggered release of 25% of encapsulated calcein as shown in Figure 3A. Similar release was achieved for 8 min pulses only after four repetitions.

Figure 3A shows that the magneto-thermally triggered release plateaued close to 50% of the encapsulated calcein set free. The passive release during the period leading to actuated release is negligible (Figure 3A; see Figure S6 for long-term passive release). However, after 5 h storage the passive release reached close to 20% with a linear release profile. The relatively high passive leakage over long time scales might be caused by per-methylation of the hydrophobic core material which was shown to render liposomes more permeable.

The PI-*b*-PNIPAM polymersomes showed reversible decrease in hydrodynamic size upon increased temperature rather than disintegration of the whole vesicles. This behavior was independent of the upper temperature (35 °C, 45 °C or 55 °C; Figures S7 and S8). In addition, for extruded vesicles, no significant change in scattering intensity or size was observed after reversible heating (Figure 3B). We therefore attribute reversible, thermally induced vesicle shrinking to a reversible partial dehydration of the interfacial PNIPAM corona that changes the membrane integrity but does not alter the vesicle topology. Thus, permeability could be increased without disassembly of the vesicles similar to what has been observed previously for polymersomes and liposomes [24,25,29].

## 3. Discussion

The control over permeability of the vesicles in this study is obtained through magneto-thermal actuation, by which an alternating magnetic field locally produces heat through its interaction with the superparamagnetic iron oxide nanoparticles embedded in the polymersome membranes. We will first discuss the principle behind the observed actuation in more detail and then compare it to a few related earlier studies on the use of magnetic hyperthermia directly on the membrane to control release from vesicles.

Magnetic relaxation and therefore local heat generation through superparamagnetic iron oxide nanoparticles can occur primarily via Néel and Brownian relaxation [37]. Néel relaxation is a universal property of magnetic materials that depends on particle core volume and refers to reorientations of the magnetic moment between different easy directions of the crystal lattice while Brownian relaxation is related to viscous rotation of the entire particle and is thus medium specific and dependent on the hydrodynamic volume. Dissipation in a restrained and viscous microenvironment like a lipid or polymer membrane is thought to preferentially proceed via Néel dissipation. Smaller particles, however, show faster spin reorientation dynamics and therefore feature faster heating rates than larger cores because the height of the rotational energy barrier is mainly associated with particle size and material anisotropy but they are also less efficient absorbers of energy from the magnetic field. The optimum size for hyperthermia of superparamagnetic nanoparticles is expected to be larger than the 3.5 nm Fe_3_O_4_ core diameter used in our vesicles. However, our attempts to embed P-NDA capped SPIONs with 8 nm cores into PI-*b*-PNIPAM block copolymer vesicles were unsuccessful. Presumably, the unfavorably high effective extension of the PI block required to encapsulate the core-shell particle in the hydrophobic core of the membrane prevented incorporation of larger particles. A redesign of the block-copolymer to higher molecular weights of both blocks could remedy this drawback. The frequency and strength of the alternating magnetic field was not optimized in our experiments. A lower field strength and a frequency tuned to the maximum of the specific absorption rate function of the SPION could be used to minimize bulk heating while maximizing the local heat generation at the cores, and therefore result in more specific and effective release.

The heat generated by the magnetic actuation of the core-shell nanoparticles diffuses to the surrounding liquid. An increased temperature exceeding the LCST of the PNIPAM corona, comprising the outer part of the polymersome membrane, leads to its partial dehydration. The resulting change in amphiphile packing parameter of the block copolymer affects membrane integrity; this is the expected cause of the increased membrane permeability that is observed in our release experiments.

This pulse length required to achieve substantial release of calcein from the polymersomes is significantly longer than required for release from liposomes with *T*_m_ comparable to the LCST of the PI-*b*-PNIPAM and with similar nanoparticles incorporated in the membrane using the same magnetic field actuation [29]. As comparison, DPPC (*T*_m_ = 41 °C) liposomes with 4% w/w loading of 3.5 nm SPION released 90% of encapsulated calcein after two 4-min pulses (unpublished data). For liposomes, the release has been demonstrated to be due to a change in membrane permeability by direct heating of the membrane by the nanoparticles without requiring bulk heating [29]. The long pulse duration necessary for triggered release from the PI-*b*-PNIPAM vesicles indicates that a thermally induced structural transition through purely local heating of the PNIPAM is not likely to have been achieved. This is further supported by the fact that the bulk temperature at the end of the AMF pulse application exceeds the temperature required for thermal transition of the polymer (*cf.* Figure 1 and Figure S6).

A maximum of 50% of the encapsulated calcein was released through magneto-thermal actuation, which given the release mechanism and the nanoscopic dimensions of the vesicles indicates that only half of the vesicles released their cargo. Since the preparation method strongly favors formation of unilamellar vesicles, as demonstrated by the OD measurements, it is likely that an inhomogeneous distribution of SPIONs between different polymersomes is the main reason for that only half of the encapsulated calcein could be released. The lower contrast of some of the small polymersomes observed in TEM (Figure 2B) could indicate low SPION loading in small vesicles and that high particle loading is required for efficient release. This, though, indicates that local actuation due to nanoparticles plays an important role in the triggered release in addition to the global temperature increase. The relative contribution of the two effects requires further experiments in which the structure and properties of the polymersome membrane are varied.

If the release rate achieved for calcein is compared to the rates previously demonstrated for membrane-associated doxorubicin purely through magnetic hyperthermia [24,25], the triggered release rates achieved here are at least twice as high and the total fraction of released compound is also higher, while the passive release is negligible and significantly lower over these time-scales than in those previous studies. This observation holds over a time-scale of a few hours, which is the time-scale foreseen for a circulating drug carrier, and which would allow for rapid release of a controlled dose.

Similar to for magnetically actuated liposomes, we observe that the release could be dosed by application of multiple pulses, realizing a major advantage of field-triggered release. Although the release behavior of our polymeric vesicles parallels that of liposome analogues during magneto-thermal actuation we note that there are fundamental differences in the underlying mechanism due to different intermolecular interactions among the constituent amphiphiles. Lipid membrane dynamics are governed by collective behavior such as lateral mobility, while polymeric assemblies are characterized by higher aggregate stability and some degree of kinetic trapping that depends on molecular weight [4]. Moreover, for magnetopolymersomes with a thermoresponsive corona, sufficient liberated heat from the SPIONs in the membrane interior needs to reach the peripheral region in order to trigger the rearrangement of the H-bonding pattern of the PNIPAM backbone; this is inherently difficult to achieve due to the high thermal conductivity of water. Although the synthesized BCP **2** is below the entanglement molecular weight of the individual blocks (M_e_(PI) ~ 6 kDa and M_e_(PNIPAM ~ 20 kDa) and thus expected to undergo Rouse diffusion, actuation primarily proceeds intramolecularly through local chain dehydration of the hydrophilic block and concomitant changes in the packing parameter [4]. In the case of liposomes with membrane-embedded SPION, the dissipated heat is released in the direct vicinity of the thermoresponsive site. The lipids undergo a phase transition due to chain melting or rotational isomerizations of the alkyl chains that alters the packing pattern of the membrane forming amphiphiles. At the phase-boundary of co-existing phases close to *T*_m_, defects exist that increase membrane permeability greatly compared to single-phase membranes above and below *T*_m_. These differences between liposomes and polymersomes make it more difficult to engineer controlled, local, triggered release through magnetically induced hyperthermia for polymersomes than for liposomes, but the generally greater versatility of physical and chemical properties of block co-polymers compared to lipids makes further optimization worthwhile.

Although our aim was to investigate the feasibility of using thermoresponsive polymersomes with well-organized SPION inserted into the membrane for magneto-thermal actuation, its primary application might be in the biomedical field. Biomedical application requires that issues of stability in biological environment and toxicology are investigated, which lie beyond the scope of this work. However, in brief, the efficient loading of SPION into the polymesome membranes is not expected to lead to toxicity due to the general low toxicity of iron oxides leading to that they are FDA approved for biomedical applications [38] Namely, iron oxides dissolve into their constituent ions in acid environments such as the lysosome, and the ions are readily absorbed by the body [39].

As is shown by the thermal tests of the polymersome stability and the magneto-thermal release tests, the vesicles are stable at body temperature and the major structural transition takes place at higher temperatures. Applicability for biological applications of the presented non-optimized model system could therefore be expected. However, it is also indicated that the passive release of the PNIPAM-stabilized polymersomes might be faster at 37 °C and another hydrophilic block with higher LCST should preferably be chosen for applications. For *in vivo* application, the potential toxicity of PNIPAM also has to be considered, and favors the exchange for another thermoresponsive polymer block. A low toxicity has however previously been demonstrated for polymersome delivery systems [40] and a cell-type dependent cytotoxicity for PNIPAM [41].

## 4. Materials and Methods

### 4.1. Synthesis

#### 4.1.1. Polyisoprene MacroRAFT Agent (1): HOOC-PI(1300)-DTB

Polyisoprene macroRAFT agent (1) was prepared as in reference [42] with slight modifications. RAFT agent (81 mg, 0.29 mmol) and AIBN (24 mg, 0.146 mmol) were weighed into a thick walled glass tube. N_2_-saturated anhydrousTHF (5.5 mL) and isoprene (6 mL, 59.9 mmol) were added and the resulting mixture was sealed under inert atmosphere. The glass tube was placed in a preheated oil bath (*T* = 125 °C) and polymerized for 2 h. The tube was then allowed to cool down to room temperature and the content was concentrated in vacuo. The resulting red-pinkish viscous oil was taken up in minimal DCM and precipitated in methanol. Compound **1** was collected by centrifugation (5000 rpm/10 min/rt), washed with methanol and dried in vacuo. Yield: 225 mg (5.5%). The macro RAFT agent was dissolved in N_2_-saturated, anhydrous dioxane at a concentration of 75 mg/mL and stored at −20°C until further use.

^1^H-NMR (300 MHz, CDCl_3_, δ): 7.98 (d, 2H, *J* = 7.5 Hz, Ph), 7.51 (t, 1H, *J* = 7.3 Hz, Ph), 7.37 (t, 2H, *J* = 7.4 Hz, Ph), 5.76 (1H, 1,2-PI), 5.12 (1H, 1,4-PI), 4.90 (2H, 1,2-PI), 4.69 (2H, 3,4-PI), 4.01 (t, 2H, *J* = 7.9 Hz, CH_2_–S–C(S)), 1.5–2.3 (CH_2_, CH_3_ PI).

UV/VIS (1,4-dioxane, λ_abs_, nm): 280 (Ph-), 296 (C=S), 334 (sh), 500 (Ph(C=S)S). The PI-block of the macro-RAFT agent was evaluated according to references [18,43] and displayed the following microstructure: 90% 1,4-addition (*cis*/*trans* ~ 2/1), 5% (1,2-addition) and 5% (3,4-addition).

#### 4.1.2. RAFT Terminated Diblock-Copolymer: HOOC-PI(1300)-*b*-PNIPAM(1000)-DTB

The thermoresponsive PNIPAM blocks were prepared similar to Shan *et al.* [44] Macro-RAFT agent **1** (1.33 mL, 75 mg/mL) was added to a solution of *N*-isopropylacrylamide (NIPAM, 1.52 g, 13.4 mmol) and AIBN (0.82 mg, 0.005 mmol) in anhydrous dioxane (6.4 mL) to yield a (M)/(macroRAFT agent) ratio of 167/1. After purging the solution with nitrogen for 20 min, the flask was immersed into a preheated oil bath (70 °C) for 20 h. After cooling down, the flask was attached to a high vacuum system to remove dioxane and sublimate residual monomer. The crude residue was washed with hot water several times and subsequently freeze-dried.

#### 4.1.3. Disulfide Terminated Diblock-Copolymer (BCP **2**): HOOC-PI(1300)-*b*-PNIPAM(1000)-SSMe (2)

Cleavage of the DTB head group was conducted according to a modified procedure of Roth *et al.* [45]. The light orange polymer was dissolved in anhydrous THF (4 mL) and mixed with *S-*methyl methanethiosulfonate (188 µL, 2.25 mmol). After purging the resulting solution with nitrogen, (2-dimethylamino) ethylamine was dropwise added via a syringe (110 µL, 1 mmol). Discoloration to a faint-yellow solution is indicative of dithioester removal and was observed within 3 h. To assure complete conversion, the reaction was allowed to stir overnight. The solution was concentrated and the residue was washed with water and methanol. After drying, the crude product was purified via silica gel column chromatography. First, residual polyisoprene was eluted using DCM/MeOH 100/1, then block copolymer **2** was obtained using DCM/MeOH 6/1 as eluent. Yield: 38 mg (21%).

^1^H-NMR (300 MHz, CDCl_3_, δ): 6.90 (1H, NH, PNIPAM), 5.76 (1H, 1,2-PI), 5.12 (1H, 1,4-PI), 4.90 (2H, 1,2-PI), 4.69 (2H, 3,4-PI), 4.00 (1H, s, C*H*(CH_3_)_2_ PNIPAM), 0.8–2.2 (CH_2_, CH_3_ PI, CH_2_, CH, PNIPAM), calculated from the M_n_ (MALDI-TOF MS) the block copolymer composition is PI_17_-*b*-PNIPAM_8.5_.

^13^C-NMR (75 MHz, CDCl_3_, δ): 174.6 (C=O, PNIPAM), 135.1 (1,4 C=C, PI), 125.0 (1,4 C=C, *cis*, PI), 124.2 (1,4 C=C, *trans*, PI), 111.2 (1,2 and 3,4 C=C, PI), 41.6 (*C*H-CO, PNIPAM), 39.8 (CH_2_, PI), 38.5 (CH_2_, PNIPAM), 32.0 (CH_2_, PI), 29.7 (CH_2_, PI), 28.3 (PNIPAM), 26.7 (CH_2_, PI), 23.5 (CH_3_, PNIPAM), 22.5 (CH_3_, 1,4-*cis*, PI), 16.0 (CH_3_, 1,4-*trans*, PI).

MALDI-TOF MS (DHB, no salt added) M_n_: 2337 g/mol, polydispersity: 1.14. For (M)/(Macro RAFT agent): 167/1 a BCP with 40 vol % PNIPAM was obtained.

ATR-FTIR (powder, cm^−1^): 3600-3200 (b, –OH), 3300 (NH, amA), 3070 (=CH_2_, 3,4-PI), 2966 (CH_3_), 2924 (CH_2_), 2874 (CH_3_), 2854 (CH_2_), 2234 (CN), 1715 ((C=O)OH), 1642 (C=O, amI + C=C, 3,4 & 1,2 PI), 1540 (NH, amII), 1453 (CH_3_, PNIPAM), 1383 (CH_3_, t-1,4-*trans* PI + PNIPAM), 1368 (CH_3_, PNIPAM), 1264 (NH, amIII), 1172, 1130 (C–C, c-1,4-*cis* PI), 1098, 1027 (=C–CH_3_, c-1,4-*cis* PI), 1004 (C–C, 3,4-PI), 909 (=CH_2_, 1,2-PI), 886 (=CH_2_, 3,4-PI), 840 (–CH=CH–, c,t-1,4-*cis,trans* PI), 690 (NH, amV), 510.

UV/VIS (MeCN, λ*_abs_*, nm): 208 (CONH), 272 (sh, -SSMe)).

### 4.2. Vesicle Formation and Release Study

#### 4.2.1. Solvent Inversion

Multilamellar vesicles (MLVs) were formed by a protocol modified from Bixner *et al*. [35]. Typically, 4 mg block copolymer were mixed with the respective weight percentage of hydrophobic SPIONs and dissolved in 200 µL THF. The mixture was dropwise added into 2 mL aqueous medium (buffer or ultrapure water) containing 5 mg/mL calcein (0.2 µm filtered) under magnetic stirring. The solvent was evaporated at room temperature under a constant N_2_ stream for 3 h and while adding Milli-Q to keep the original total volume. The as-prepared vesicle suspension was extruded 10-times through 100 nm track-etched polycarbonate membranes in a hand-held extruder (Avanti) to increase the encapsulation efficiency and improve lamellarity.

#### 4.2.2. Release Assays

Removal of non-encapsulated dye and free nanoparticles from the extruded samples was performed on a Bio Logic DuoFlow chromatography system equipped with a UV-detector (Bio-Rad, Austria), a Knauer Smartline RI 2300 detector (Bio-Rad, Austria) and a Bio Logic BioFrac collector (Bio-Rad, Austria). In detail, the samples (2 mL, 2 mg/mL) were purified by passing over a FPLC-column (length × diameter: 60 cm × 3 cm, stationary phase: Superdex 75, (GE Life Sciences, Austria) in Milli-Q water with a flow rate of 0.75 mL/min. Fractions of 2 mL containing the desired sample (usually 4 fractions) were identified by UV and RI detection. The sample concentration decreased to 0.5 mg/mL by the purification process.

#### 4.2.3. Magnetic Actuation

The as-prepared sample was filled in a PMMA cuvette which was placed in an Ambrell Easy Heat LI magnetic heater, with a current of 438.9 A and a frequency of 228 kHz, coil dimension (height × outer diameter × coil thickness × number of turns = 37 mm × 37 mm × 2 mm × 6). The field strength in the center of the coil where the sample is placed is with these parameters estimated to be ~95 mT. Magnetic actuation was performed in 8 min or 10 min cycles, with a delay of 5 min between the cycles for recording of the released amount of calcein via fluorescence spectroscopy.

#### 4.2.4. Fluorescence Measurements

Fluorescence spectra were collected with a PerkinElmer LS 55 luminescence spectrometer (PerkinElmer, Austria) at an excitation wavelength of 495 nm and an emission wavelength of 515 nm with a scan speed of 100 nm/min and a slit width of 2.5 nm. In some cases, the sample was diluted further in order to be within the optimal working range of the photo detector. Release of calcein was calculated according to the formula:

(1) $$Release\%=\frac{I_{i}-I_{AMF}/I_{\;PL}}{I_{i}-I_{tot}}$$

where *I_i_* is the initial fluorescence intensity measured immediately after column purification, *I_AMF_* is the fluorescence intensity measured after the sample was subjected to individual AMF treatments and *I_PL_* is the fluorescence intensity measured at different times without applying any AMF in order to calculate passive leakage. *I_tot_* is the total fluorescence intensity measured after complete lysis of the vesicles by addition of Triton X100 (10% v/v of 20% Triton in MQ water).

### 4.3. Methods

#### 4.3.1. TEM

TEM studies were performed on a FEI Tecnai G2 20 transmission electron microscope (FEI, Austria) operating at 160 kV. Samples were prepared by dropping aqueous vesicle dispersions containing 1%–3% w/w (+)-d-trehalose onto 300-mesh carbon-coated copper grids and subsequently air drying the samples for a few hours.

#### 4.3.2. Dynamic Light Scattering

Hydrodynamic diameters, aggregation points and temperature cycling experiments were measured on a Malvern Zetasizer Nano-ZS (Malvern Instruments, Germany) in Milli-Q water at variable temperature in 173° backscattering mode. Samples were equilibrated for 120 s each and the autocorrelation function was obtained by averaging 3 runs. Samples were measured at 100 µg/mL.

#### 4.3.3. TGA/DSC Measurements

Thermograms were recorded on a Mettler-Toledo TGA/DSC 1 STAR System (Mettler Toledo GmbH, Vienna, Austria) in the temperature range 25–650 °C with a ramp of 10 K/min under 80 mL/min synthetic air gas flow. The mass loss was evaluated by horizontal step setting.

#### 4.3.4. ^1^H- and ^13^C-NMR Measurements

^1^H and ^13^C-solution spectra were collected on a Bruker AV III 300 spectrometer (Bruker Austria GmbH, Vienna, Austria). Chemical shifts were recorded in ppm and referenced to residual protonated solvent (CDCl_3_: 7.26 ppm (^1^H), 77.0 ppm (^13^C)).

#### 4.3.5. MALDI-TOF-MS Measurements

Mass spectra were collected using a Bruker Autoflex (Bruker, Austria) speed in reflector positive mode (laser power: 20%, number of shots: 30,000). Matrix: DHB (20 mg/mL) in THF was mixed with sample and dropped on a sample holder. Calibration was performed with Peptide Calibration Standard II from Bruker (Bruker, Austria).

#### 4.3.6. ATR-FTIR Measurements

Mid-IR powder spectra of the lyophilized samples were collected using a Bruker Tensor 37 FTIR spectrometer with a Bruker Platinum Diamond single reflection ATR equipment (Bruker, Austria) at a resolution of 4 cm^−1^ by averaging 32 scans.

#### 4.3.7. UV-Vis Measurements

UV-Vis absorption spectra were collected at a scan speed of 400 nm/min on a Hitachi UV-2900 spectrophotometer (Hitachi Power Tools, Austria).

## 5. Conclusions

In summary, we have demonstrated synthesis and assembly of PI-*b*-PNIPAM block copolymersomes into which well-stabilized hydrophobic core-shell SPIONs can be incorporated. The size and stability of the assemblies are suitable for nanoscale encapsulation and release applications. A hydrophilic compound could be released by magnetic heating that induced a reversible structural change in the polymersome membrane. The release was controlled but not as efficiently as previous demonstrations of similar release strategies using liposomes; this could possibly be improved by optimizing the structure using a higher molecular weight block copolymer for which the hydrophilic block undergoes a more drastic volumetric change upon dehydration than is the case for short PNIPAM blocks. A correspondingly higher MW hydrophobic block also allows for incorporation of large SPIONs that provide more efficient heating. Compared to previously demonstrated release of membrane associated compounds from polymersomes using magnetically induced hyperthermia, the release rate of our hydrophilic model compound was higher and the passive release over relevant time scales significantly lower. Further development of the introduced structure and mechanism for magneto-thermal release from polymersomes here could result in a delivery system optimized for advanced, dosed release in, for example, the biomedical field.

## Supplementary Materials

The following are available online at www.mdpi.com/1996-1944/9/1/29/s1.
