# Supplementary material for: Triggered Release from Thermoresponsive Polymersomes with Superparamagnetic Membranes
Source: Materials (Basel). 2016 Jan 6;9(1):29. doi: 10.3390/ma9010029 (PMC5456531; doi:10.3390/ma9010029)
Supplement: Supplementary file 1 [file materials-09-00029-s001.pdf]

Supplementary Materials: Triggered Release from Thermoresponsive Polymersomes with Superparamagnetic Membranes

Oliver Bixner, Steffen Kurzhals, Mudassar Virk and Erik Reimhult

1. Reagents

All chemicals were purchased from Sigma-Aldrich (Austria) and used without further purification except otherwise noted.

4-cyano-4-(phenylcarbonothioylthio)pentanoic acid > 97%; isoprene 99% (contains < 1000 ppm *p*-*tert*-butylcatechol as inhibitor); *N*-isopropylacrylamide 97%; 2,2'-azobis(2-methylpropionitrile) 98%; *S*-methyl methanethiosulfonate 97%; N,N-dimethylethylenediamine 95%; Milli-Q water (R = 18 MΩ·cm); methanol anhydrous ≥ 99.8%; acetone chromasolv for HPLC ≥ 99.9%; dichloromethane anhydrous ≥ 99.8% (contains 50–150 ppm amylene as stabilizer); chloroform ≥ 99.5% (containing 100–200 ppm amylenes as stabilizer); tetrahydrofuran chromasolv plus for HPLC ≥ 99.9% (inhibitor-free); 1,4-dioxane ACS reagent ≥ 99.0%; toluene anhydrous 99.8%; n-hexane anhydrous 95%; aluminum oxide activated, basic, Brockmann I (150 mesh); (+)-D-trehalose dihydrate from corn starch > 99%.

N-isopropylacrylamide (NIPAM) was recrystallized from hexane/toluene: 1/1 v/v. 2,2'-azobis(2-methylpropionitrile) (AIBN) was recrystallized from methanol. Isoprene was purified by passing through a column of basic alumina.

2. Spectroscopic Analysis

Figures S1–S3 show additional characterization of the synthesized block copolymer BCP **2**, demonstrating purity of the product.

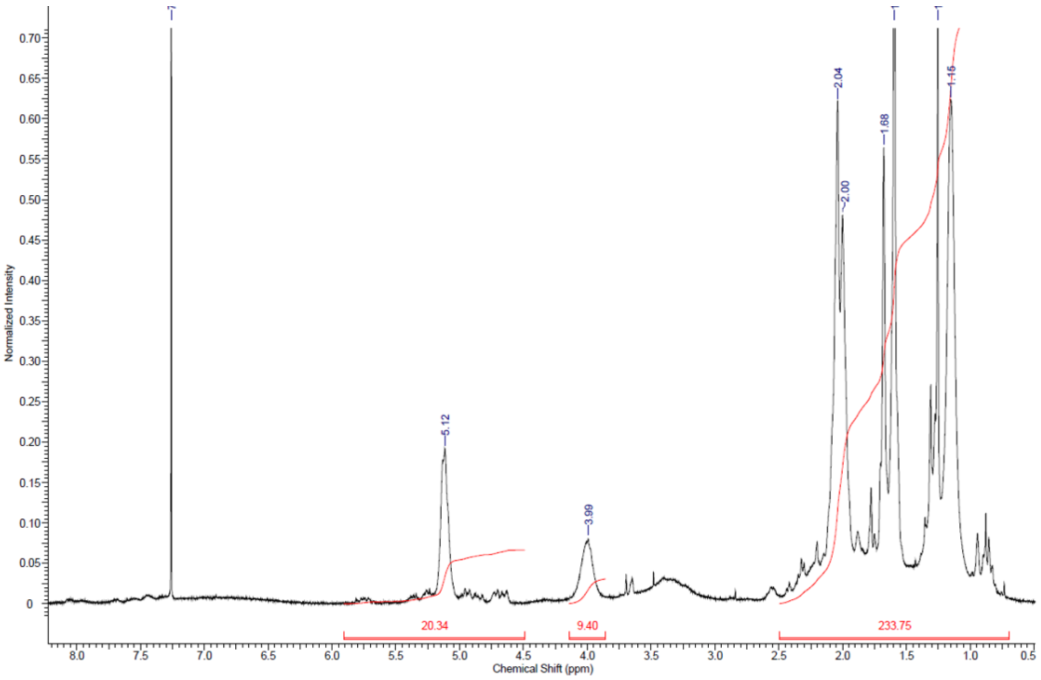

Figure S1. ^{1}H-NMR spectrum of BCP **2** in $CDCl_3$.

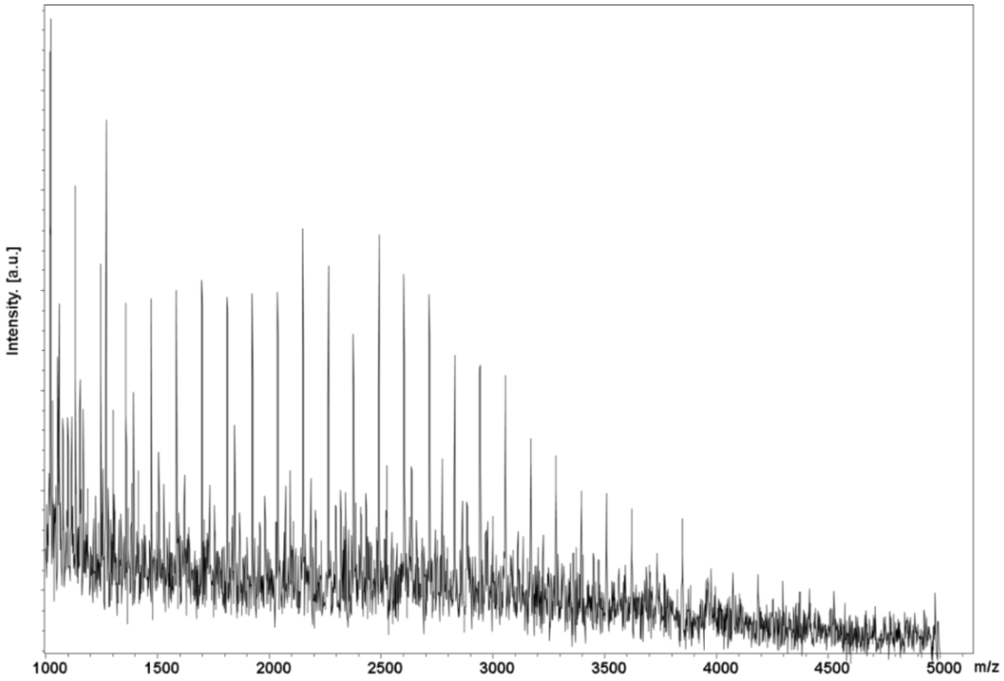

Figure S2. MALDI-TOF mass spectrum of BCP **2**, DHB, no salt added, 1000–5000 Da, reflector positive mode.

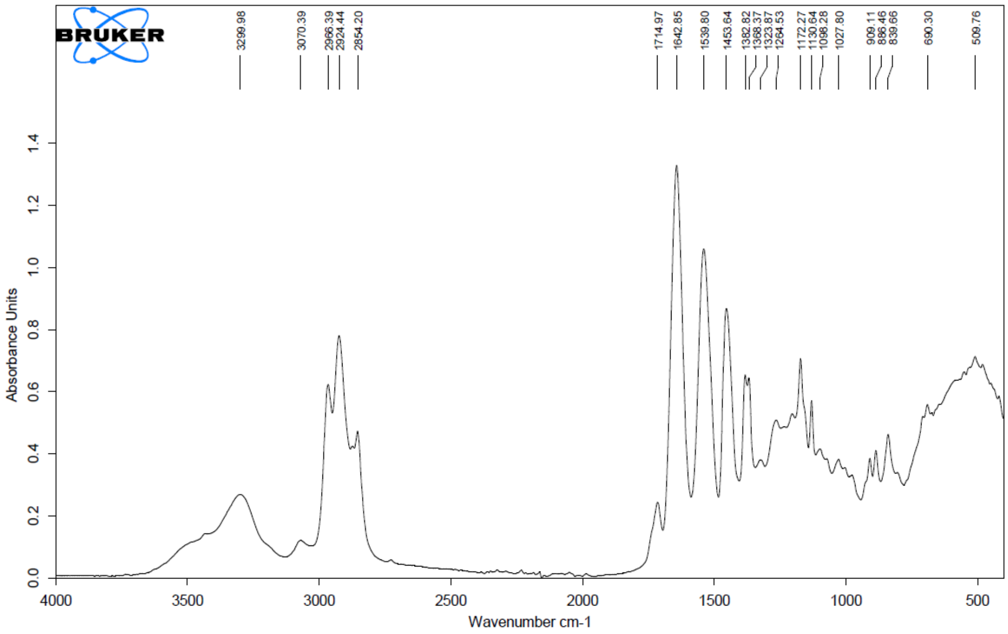

Figure S3. ATR-FTIR spectrum of lyophilized BCP **2**.

3. Characterization of Residual THF

THF remaining at high concentration in the sample could affect stability over time and lead to toxicity in biological applications. However, THF is a high vapor pressure solvent and is readily evaporated under continuous nitrogen flow until a homogeneous suspension of vesicles containing SPIONs is achieved. The amount of residual THF for the preparation of liposomes through the same protocol for solvent inversion was previously quantified by us by NMR to be 0.05 ‰ or 50 ppm of its initial value (Figure S4) [1]. Also after 3 h under continuous nitrogen evaporation it is negligible. Such minimal traces of THF retained are far below any toxic level and suitable for biological and medical applications [2].

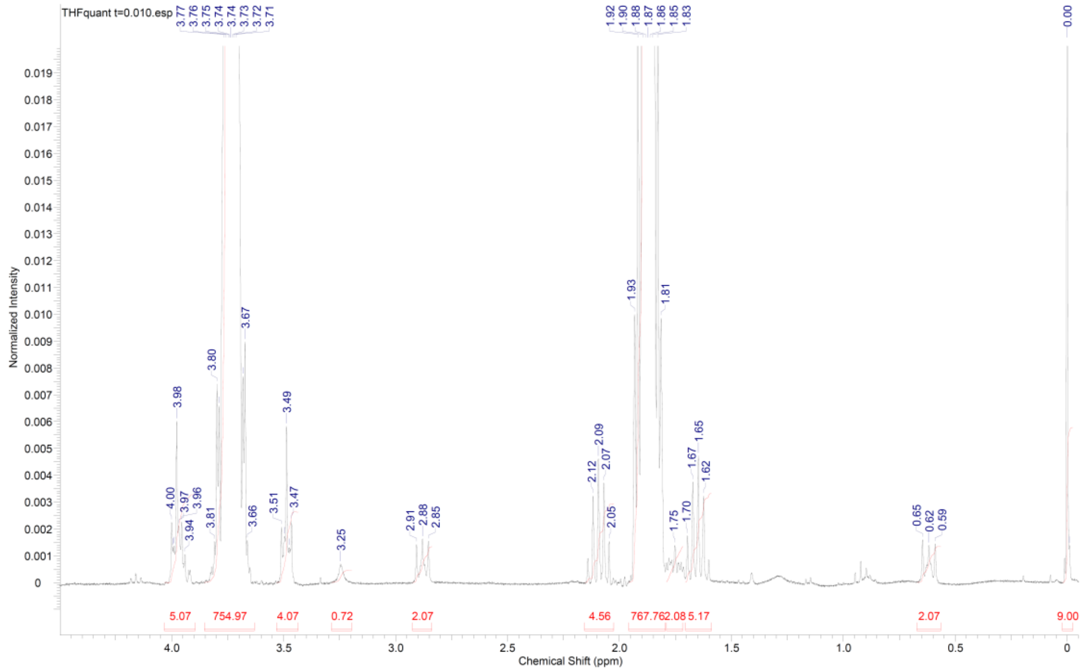

(**A**)

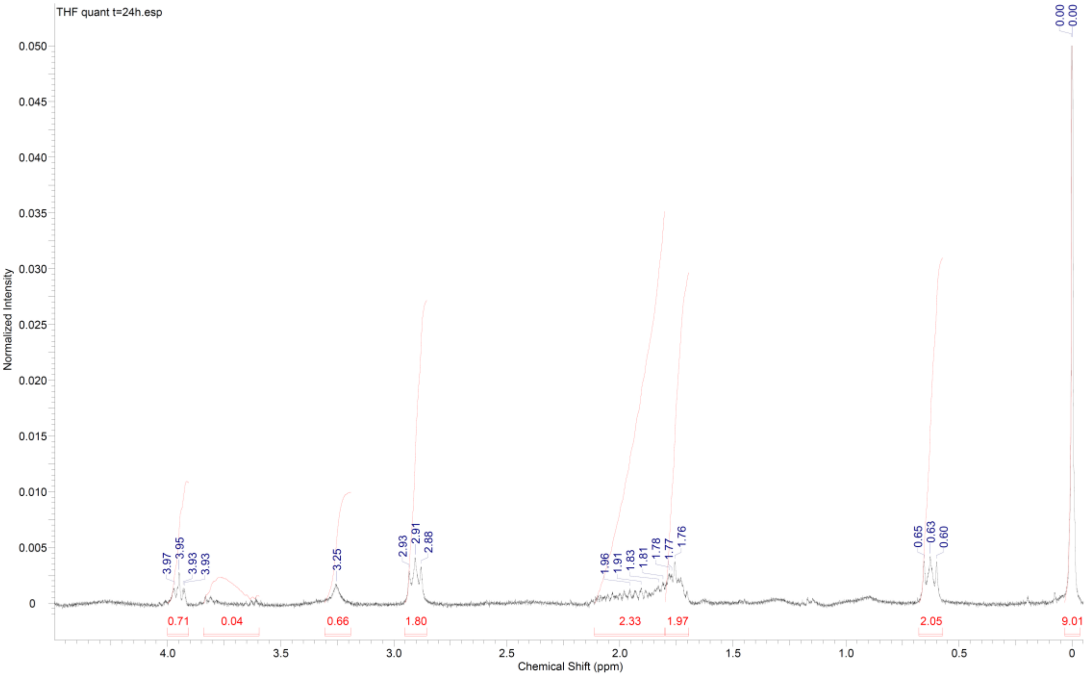

(**B**)

Figure S4. ^{1}H NMR spectra (300 MHz) of POPC in D_2O containing 1 mg/mL DSS as reference standard. Liposomes were formed at 0.5 mg/mL via 1:10 solvent inversion. (**A**) NMR spectrum right after dropwise addition of THF at t = 0 h and (**B**) after 24 h of evaporation. Residual THF was quantified by integration of the resonances at δ = 3.74 (CH_2O) and 1.87 ppm (CH_2).

4. Determination of the Iron Oxide Nanoparticle Loading

4.1. Additional TEM of SPION-Loaded Polymersomes

Figure S5 shows additional TEM micrographs of calcein-loaded, extruded PI-*b*-PNIPAM polymersomes with 20% w/w 3.5 nm hydrophobic SPION input.

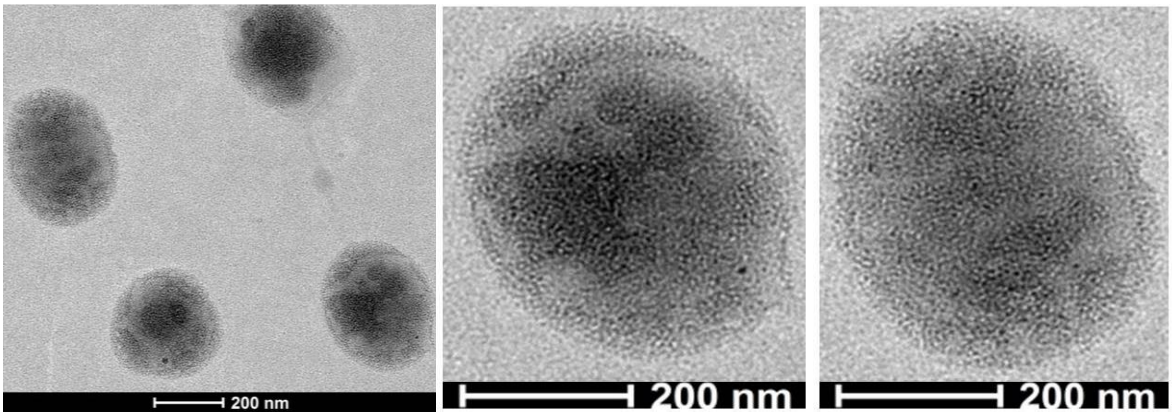

Figure S5. TEM micrographs of calcein-loaded, extruded PI-*b*-PNIPAM polymersomes at 1 mg/mL with 20% w/w 3.5 nm hydrophobic SPION input. Samples were prepared by THF solvent inversion into 5 mg/mL calcein solution to form polydisperse, large polymersomes and subsequent extrusion through 100nm track-etched polycarbonate membranes after evaporation of the organic solvent.

4.2. Thermogravimetric Analysis

For TGA determination of the effective SPION content of the polymersome membranes the lyophilized samples were burnt under oxidative conditions (synthetic air) to yield near complete combustion. Yet a considerable residue (~11% w/w) remained even in the case of polymersomes containing no SPIONs. The reported final SPION loading content therefore refers to the non-combusted material at 650 °C in excess of the residue for samples not containing nanoparticles, which amounts to approximately 9% w/w for extruded SPION loaded samples.

4.3. Optical Density Determination

Optical density (OD) values at 350 nm (OD^{350}) were used for spectroscopic quantification of the SPION embedding efficiency. The OD^{350} values were obtained by dilution of the respective suspensions to match the amide absorptions at 208 nm. Background spectra of the plain extruded PI-*b*-PNIPAM vesicles were recorded to account for vesicular scattering. The OD^{350} value of the initial SPION loaded suspension was assigned to the input SPION weight fraction (20%) and the final loading content was determined by evaluating the OD^{350} decrease upon extrusion. In this way we estimate an effective loading content of around 9% w/w which agrees with the results obtained by TGA. The slight difference between the two methods is likely to be caused by remote mass loading and use of augmenting relations in TGA while the choice of evaluation wavelength and variations in lamellarity cause deviations in UV/VIS.

5. Characterization of SPION Loaded, Extruded Assemblies of BCP 2

Figure S6A shows the increase in bulk temperature resulting from magnetic heating and Figure S6B shows the long-term passive release of calcein at room temperature when no actuation is applied. Figures S7 and S8 show the effect of temperature on polymersome size from bulk heating and from localized heating by application of an alternating magnetic field.

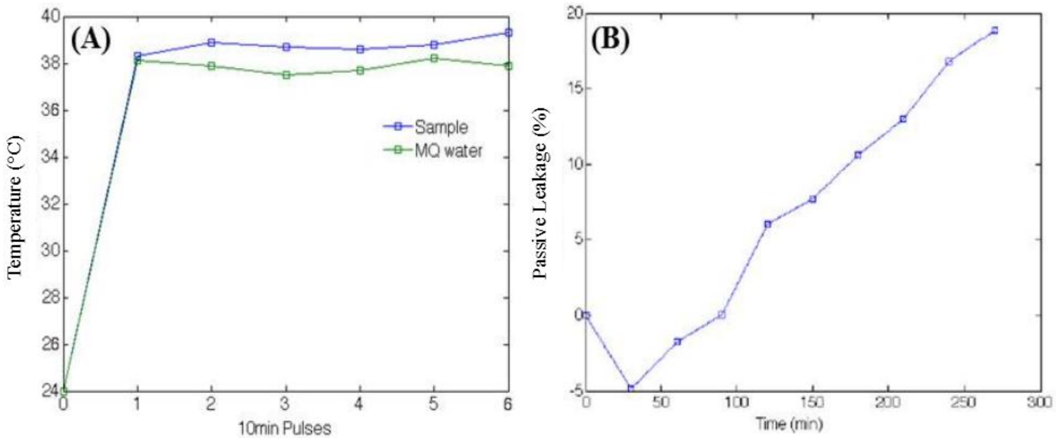

Figure S6. (**A**) Bulk temperatures upon magnetic heating for SPION-loaded samples (blue) and ultrapure water (green) as reference; (**B**) Passive leakage of the vesicles evaluated over a 5 h time course at room temperature (24 °C) shows linear release of encapsulated calcein. Over a time period equivalent to that of the complete magnetic actuation sequence there is no release observed.

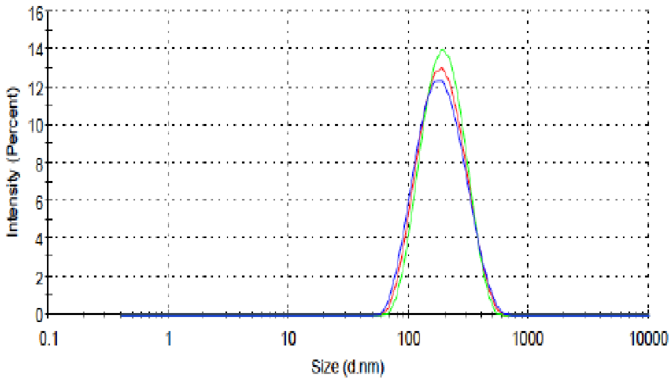

(**A**)

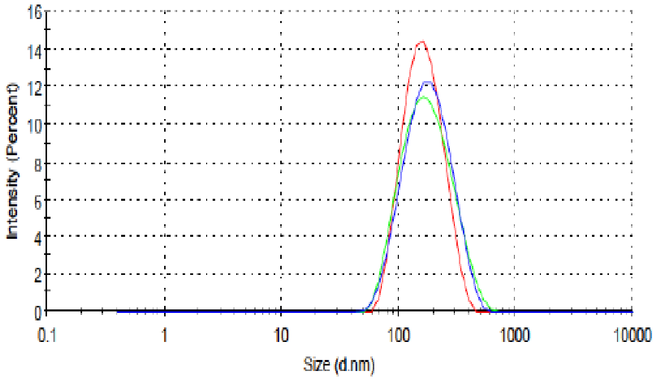

(**B**)

Figure S7. DLS size distributions of SPION-loaded polymersomes before (**A**) and after (**B**) AMF heating.

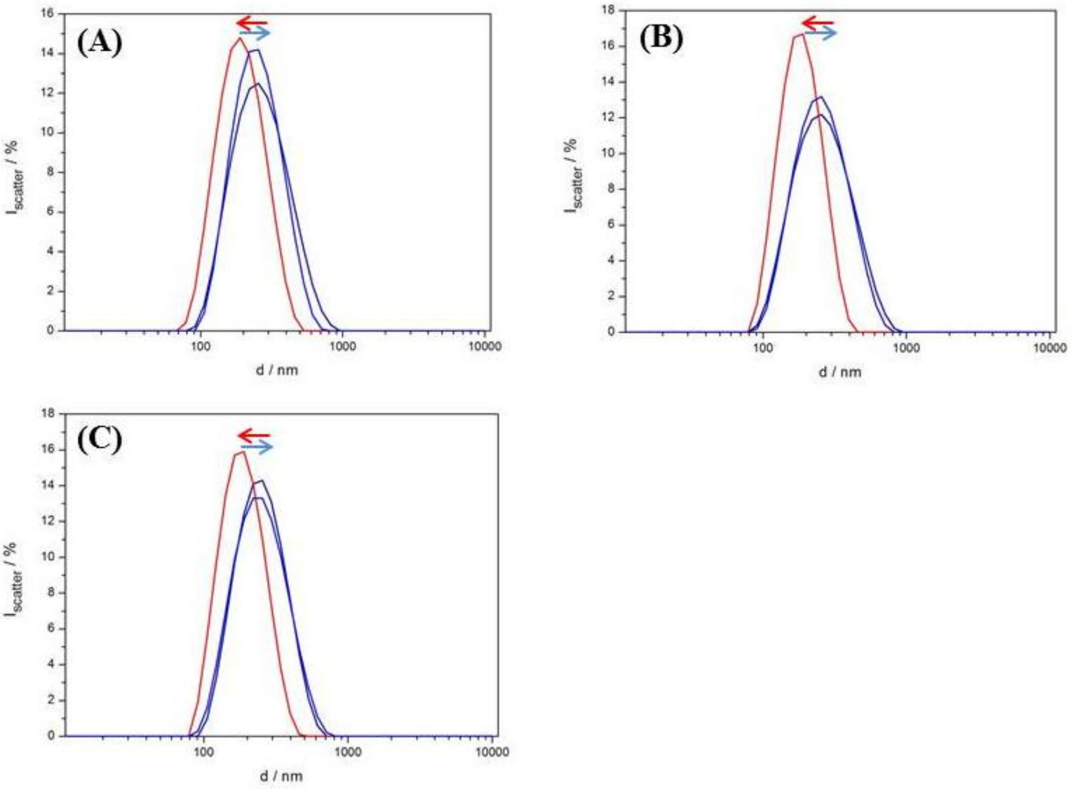

Figure S8. DLS size-distributions of the thermoresponsive polymersomes during temperature jumps (**A**) 25 °C–35 °C–25 °C; (**B**) 25 °C–45 °C–25 °C and (**C**) 25 °C–55 °C–25 °C exhibiting reversible size changes of the vesicles without signs of disintegration.

References

1. Bixner, O.; Reimhult, E. Controlled magnetosomes: Embedding of magnetic nanoparticles into membranes of monodisperse lipid vesicles. *J. Colloid Interface Sci.* **2016**, *466*, 62–71.
2. Fowles, J.; Boatman, R.; Bootman, J.; Lewis, C.; Morgott, D.; Rushton, E.; van Rooij, J.; Banton, M. A review of the toxicological and environmental hazards and risks of tetrahydrofuran. *Crit. Rev. Toxicol.* **2013**, *43*, 811–828.
